# Uptake Patterns of Glycine, Ammonium, and Nitrate Differ Among Four Common Tree Species of Northeast China

**DOI:** 10.3389/fpls.2019.00799

**Published:** 2019-07-02

**Authors:** Feifei Zhu, Luming Dai, Erik A. Hobbie, Keisuke Koba, Xueyan Liu, Geshere A. Gurmesa, Shaonan Huang, Shanlong Li, Yinghua Li, Shijie Han, Yunting Fang

**Affiliations:** ^1^CAS Key Laboratory of Forest Ecology and Management, Institute of Applied Ecology, Chinese Academy of Sciences, Shenyang, China; ^2^Qingyuan Forest CERN, Shenyang, China; ^3^School of Resources and Civil Engineering, Northeastern University, Shenyang, China; ^4^Earth Systems Research Center, Morse Hall, University of New Hampshire, Durham, NH, United States; ^5^Center for Ecological Research, Kyoto University, Shiga, Japan; ^6^Institute of Surface-Earth System Science, Tianjin University, Tianjin, China; ^7^College of Resources and Environment, University of Chinese Academy of Sciences, Beijing, China; ^8^School of Life Sciences, Henan University, Kaifeng, China

**Keywords:** glycine, ammonium, nitrate, nitrogen uptake, nitrogen availability, stable isotopes, secondary forest in northeast China

## Abstract

Fundamental questions of how plant species within secondary forests and plantations in northeast China partition limited nitrogen (N) resource remain unclear. Here we conducted a ^15^N tracer greenhouse study to determine glycine, ammonium, and nitrate uptake by the seedlings of two coniferous species, *Pinus koraiensis* (*Pinus*) and *Larix keampferi* (*Larix*), and two broadleaf species, *Quercus mongolica* (*Quercus*) and *Juglans mandshurica* (*Juglans*), that are common in natural secondary forests in northeast China. Glycine contributed 43% to total N uptake of *Pinus*, but only 20, 11, and 21% to N uptake by *Larix, Quercus*, and *Juglans*, respectively (whole plant), whereas nitrate uptake was 24, 74, 88, and 68% of total uptake for these four species, respectively. Retention of glycine carbon versus nitrogen in *Pinus* roots indicated that 36% of glycine uptake was retained intact. Nitrate was preferentially used by *Larix, Quercus*, and *Juglans*, with nitrate uptake constituting 68∼88% of total N use by these three species. These results demonstrated that these dominant tree species in secondary forests in northeast China partitioned limited N resource by varying uptake of glycine, ammonium and nitrate, with all species, except *Pinus*, using nitrate that are most abundant within these soils. Such N use pattern may also provide potential underlying mechanisms for the higher retention of deposited nitrate than ammonium into aboveground biomass in these secondary forests.

## Introduction

In Northeast China, natural secondary forests (NSFs) account for as much as 70% of the regional forests (Hao et al., 2000). They are formed through natural regeneration of primary forests following afforestation (Zhu and Liu, 2007). Since the 1950s, large areas of the NSFs have been turned into larch plantations for fast timber production (Wang et al., 2006), resulting in mosaic plantation/secondary forest landscapes. These two forest types are common in China and are regionally important in providing ecosystem services of regulating regional climate. However, several problems have emerged, including soil acidification and nutrient leaching from these disturbed forest sites (Liu et al., 1998; Yang et al., 2010). Relatively lower soil nutrient concentrations, especially available nitrogen, in these secondary forests and larch plantations compared to those well-developed and less-disturbed forests may constrain tree growth (Yang et al., 2012). However, fundamental questions of how plant species within these forests partition and compete for limited soil N resource remain unclear.

A complete understanding of forest N cycling requires that we understand how much N plants take up, and which form of inorganic or organic N they assimilate. Soil N occurs in a variety of organic and inorganic forms that are differentially available to plant roots. Complex organic polymers in litter are depolymerized into bioavailable, N-containing monomers (such as amino acids, Schimel and Bennett, 2004) that are then mineralized by microorganisms, producing ammonium (${\mathrm{NH}}_{4}^{+}$) and nitrate (${\mathrm{NO}}_{3}^{–}$). Plant species differ in their capacity to take up ammonium, nitrate or organic N as their primary N source partly because of physiological trade-offs in taking up different N forms (Stewart et al., 1992). While nitrate is readily available due to its high solubility in soil water, plants must use energy to reduce nitrate before incorporation into amino acids. Thus, it may be more efficient for plants to take up ammonium because it can be immediately incorporated into amino acids. However, ammonium is not always available for uptake due to its strong adsorption to soil exchange sites and poor mobility in soil solution. Free amino acids, which are found in large quantities in some ecosystems, could also be a major N source for plant due to their ready incorporation into plant protein (Persson and Näsholm, 2001; Näsholm et al., 2009). On the other hand, nitrate is often faster transported out of roots than amino acids or ammonium, bypassing the reducing and assimilation steps in the roots (Persson et al., 2006; Scott and Rothstein, 2011).

In addition, plants differ in their N form preference. For example, early and late successional tree species had different uptake rates for nitrate versus ammonium, respectively, which were related to the availability of these N forms along successional gradients (Kronzucker et al., 1997). Conifer trees may not grow well in disturbed sites because of their preference for ammonium and their poor ability to use nitrate that often dominates disturbed sites (Ingestad, 1979; Kronzucker et al., 1997). In N-limited arctic and alpine tundra plant communities, species composition correlated with the partitioning of differentially available forms of N, with dominance being linked to the ability of a species to exploit the most abundant N form (McKane et al., 2002; Ashton et al., 2010). On the other hand, plants are often flexible in their capacity to take up the chemical forms of N that are most readily available (Scott and Rothstein, 2011). For example, based on ^15^N natural abundance in vegetation and soils, tree species in Hawaiian tropical forests along a rainfall gradient did not specialize on different soil N pools, but rather responded to changes in soil N availability to exploit the most abundant N form (Houlton et al., 2007). In the tropical montane forest in Panama, ^15^N tracer studies indicated flexibility in uptake of N forms in tropical plants across root trait groups, with only a few species (5 out of 11) weakly preferring specific N forms (Andersen et al., 2017). Whether plants within forests prefer specific N forms or are plastic in their response is important to understand because variability in N uptake implies variability in N retention within forested watersheds.

Most previous studies on uptake of different forms of N by temperate tree species used hydroponic experiments, in which either intact or excised roots were submerged in ^15^N tracer solutions and the ^15^N enrichment within roots determined after several hours (Warren and Adams, 2007; Liu et al., 2017, references cited therein). These studies demonstrated the inherent uptake capacity of tree species for different N forms, reflecting the functional niche of specific species within an ecosystem (McKane et al., 2002). Using this method, Liu et al. (2017) demonstrated that tree species in temperate forest in Donglingshan, China, prefer ammonium, with ammonium uptake accounting for 76% of the total N uptake of these tree species, including *Juglans mandshurica* Maxim. Other studies directly labeled the soil in pots (Templer and Dawson, 2004) or in the field (McKane et al., 2002; Persson et al., 2003; Gallet-Budynek et al., 2009; Andersen et al., 2017) and studied ^15^N incorporation into whole plants. In these studies, plants either preferred different N forms, with dominant plants using the most available forms of N within local soil (McKane et al., 2002; Templer and Dawson, 2004; Gallet-Budynek et al., 2009), or plants took up all N provided, regardless of N form (Persson et al., 2003; Andersen et al., 2017).

In this study, we selected two coniferous species, *Pinus koraiensis* and *Larix keampferi* and two broadleaf species, *Quercus mongolica* and *Juglans mandshurica*, that are common in NSFs and larch plantations in northeast China to study their uptake of different N forms. We used both ^15^N natural abundance (δ^15^N) methods and a ^15^N tracer experiment with ammonium, nitrate and glycine to determine if these tree species show preference or flexibility in N use. Glycine is the most frequently used amino acid in field studies of plant uptake of amino acids (Jones et al., 2005) because of its high abundance in forest soils (Finzi and Berthrong, 2005; Berthrong and Finzi, 2006; Liu et al., 2017), mobility in the soil (Owen and Jones, 2001) and poor substrate quality for microbial growth (Lipson et al., 1999). We, on one hand, used isotope mixing models to calculate the contributions of soil dissolved organic N (DON), ammonium and nitrate to plant N use by these four tree species based on the δ^15^N values of different soil N pools and δ^15^N signature of plant leaves (Houlton et al., 2007; Takebayashi et al., 2010; Liu et al., 2018). In addition, we labeled tree seedlings of these four species growing in pots in greenhouses with ^15^N tracer to determine short-term uptake and assimilation pattern of glycine, ammonium, and nitrate. We hypothesized that (1) the two coniferous species *Pinus koraiensis* and *Larix keampferi* will prefer ammonium or glycine to nitrate (Kronzucker et al., 1997); (2) the two broadleaf tree species *Quercus mongolica* and *Juglans mandshurica* will prefer nitrate since nitrate is often the dominant inorganic N form in disturbed forest sites (Hope et al., 2003); and (3) the N uptake patterns of these tree species should be related to the availability of these N forms in their natural habitat.

## Materials and Methods

### Species Selection and Seedling Preparation

We selected four common tree species in the NSF near Qingyuan Experimental Station of Forest Ecology of the Institute of Applied Ecology, Chinese Academy of Sciences, which is located in a mountainous region in the eastern Liaoning Province, northeast China (41°51′ N, 124°54′ E, elevation 500–1100 m above sea level). These species include two coniferous species, *Pinus koraiensis* and *Larix keampferi*, and two broadleaf species, *Quercus mongolica* and *Juglans mandshurica*, which make up 5, 30, 8, and 27% of the mature tree composition in this forest, respectively (forest survey, Li et al., unpublished data). These four species are known to be associated with different mycorrhizal types, with *Juglans mandshurica* associated with arbuscular mycorrhizal fungi, with the three other species, *Pinus koraiensis, Larix keampferi*, and *Quercus mongolica* all being ectomycorrhizal type (Guo et al., 2008; Liu et al., 2017). The continental monsoon climate of the region has a humid and rainy summer and a cold and snowy winter. Mean annual air temperature varies between 3.9 and 5.4°C and ranges from -37.6 to 36.5°C. The mean annual precipitation ranges between 700 and 850 mm. 80% of the rain falls in June, July, and August. The current wet N deposition rate in this forest is 19 kg N ha^-1^ yr^-1^ (mean value during 2014 to 2017), and the ratio of ammonium to nitrate is 2:1 (Huang et al., 2019). The soil type is a typical brown forest soil with a thickness of 60–80 cm.

One-year old seedlings of these four species were purchased from a local nursery and transplanted to the greenhouse in Yunong Campus, Institute of Applied Ecology, Chinese Academy of Sciences (41°90′ N, 123°58′ E). Soils for transplanting these seedlings were topsoils from low hills adjacent to the greenhouse, which are dominated by *Pinus koraiensis, Ulmus laciniata*, and were classified as brown forest soils, with pH of 6. The seedlings were transplanted individually to each pot (15.3 L). All pots received natural light levels and were watered every 3 days with tap water to keep the soil surface moist. The average high temperature in the greenhouse during the experiment was 26.5°C and the average low temperature was 18.3°C. Pots of all seedlings were arranged randomly and were moved within the greenhouse regularly to reduce the effects of environmental variation. These seedlings were acclimated to greenhouse conditions for 1 year prior to the ^15^N labeling experiment. No nutrients were added to the pots during this 1 year acclimation.

### ^15^N Labeling Experiment

Most leaves reached full expansion by middle May. We labeled each individual seedling with ^15^N tracers in July 2018. For ^15^N labeling, we randomly allocated four individuals of each species to one of the three N species treatments (U-^13^C_2_, ^15^N-glycine, ^15^NH_4_Cl, or Na^15^NO_3_). Seedlings received 10.17 mg ^15^N per individual (100 mg ^15^N m^-2^, equal to 0.34 μg ^15^N g^-1^) dissolved in 20 mL deionized water containing equimolar concentrations of nitrate, ammonium, and glycine, with only one N form labeled. The total amount of N (including ^15^N) added to each plant individual was 1.02 μg N g^-1^soil, with 1/3 ^15^N, representing a roughly 16∼37% increase in DIN (ammonium+nitrate) and a 8∼14% increase in total N. The organic N source was provided as U-^13^C_2_, ^15^N-glycine (both ^13^C and ^15^N at 99 atom%), while ammonium and nitrate were supplied as ^15^NH_4_Cl (99.08 atom%) and Na^15^NO_3_ (99.26 atom%). We could then test for uptake preferences of each of the labeled N forms by individual plant species and also test the proportion of glycine taken up directly by plants, as evidenced by the relative enrichment of plant tissue with ^13^C and ^15^N, or as mineral N after microbial mineralization (Bardgett et al., 2003). The nitrification inhibitor dicyandiamide (DCD) was added at 50 μg g^-1^ to all three ^15^N labeling solutions to prevent potential nitrification of ammonium. In a preliminary experiment, 50 μg g^-1^ of the nitrification inhibitor DCD effectively inhibited nitrification over 48 h under *Pinus* soil (data not shown). A control group with no added N provided background ^15^N values for ^15^N uptake rates calculations.

We removed any leaf litter within the surface of the pots and injected nitrogen tracer solutions into the soil at four locations (5 ml for each) equally spaced around, and approximately 3 cm from, the stem of each seedling to a depth of 10 cm using a syringe and needle with four lateral holes. After 24 and 48 h, seedlings and intact root systems were harvested and bulk soil surrounding each seedling (∼3 cm radius or within the ^15^N-label zone) was collected for analysis. Seedlings were immediately rinsed, separated into coarse roots (>2 mm), fine roots (<2 mm), stems, and leaves, dried at 60°C for 48 h, weighed, and ground for ^15^N and ^13^C isotope analyses by elemental analyzer-isotope ratio mass spectrometry (Elementar AnalysenSysteme GmbH, Germany; IsoPrime100, IsoPrime Limited, United Kingdom). Four compounds were used as references: L-histidine, D-glutamic acid, glycine, and acetanilide. The analytical precision for δ^15^N and δ^13^C were both 0.2‰. Whole plant δ^15^N values were calculated from mass-weighted δ^15^N values of each plant organ.

^15^N atom% excess (APE) was calculated as the difference in atom% ^15^N between ^15^N-labeled plant organs and unlabeled (control) organs. ^15^N uptake rates (μg ^15^N g^-1^ dry mass h^-1^) were calculated by multiplying plant organ N content (μg g^-1^ plant organ biomass) by the corresponding (APE/100), divided by labeling time in hours and (atom% ^15^N/100) of the applied ^15^N-labeled N form (99 atom% for U-^13^C_2_, ^15^N-glycine, 99.08 atom% for ^15^NH_4_Cl, 99.26 atom% for Na^15^NO_3_) as follows: (N content (μg g^-1^ plant organ biomass) × APE/100)/(time (h) × (at% ^15^N tracer/100)) (Liu et al., 2017). Uptake rates of each N form were normalized to the sum of uptake rates of the three N forms to calculate its contribution to total N uptake or assimilation. Here we only report results of 48 h after N tracer addition, since preliminary results showed 48 h to be necessary to detect obvious ^15^N enrichment in plant leaves (data not shown).

### Soil Chemical and Isotopic Analyses

Total dissolved N (TDN), ammonium, and nitrate were immediately extracted within 4 h of sampling in 50 mL of 2 mol/L KCl on a shaker table for 1 h, filtered through Whatman GF/A filters (Whatman PLC, Maidstone, United Kingdom), and frozen at -36°C until colorimetric determination of nitrate+nitrite and ammonium using a continuous chemical analyzer (SmartChem 200, Rome, Italy). Gravimetric soil water content was 66 ± 9.7% on average at the time of harvest. Total dissolved N in KCl extracts was determined by alkaline persulfate digestion (Cabrera and Beare, 1993; Doyle et al., 2004) and nitrate detection by continuous chemical analyzer (SmartChem 200, Rome, Italy).

The δ^15^N measurements of actual and derived nitrate samples were then made using the modified azide method (Tu et al., 2016). Methodological details, including standard preparation, were detailed previously (Tu et al., 2016). The δ^15^N values of KCl-extracted ammonium were analyzed using the acidified disk diffusion method to isolate ammonium from the remainder of the KCl-extracted TDN (Stephan and Kavanagh, 2009). The δ^15^N value of dissolved organic N (δ^15^N_DON_) was calculated as the mass-weighted difference between the concentrations of KCl-extracted TDN, ammonium, and nitrate, and the δ^15^N values obtained from the modified azide method (oxidized TDN and actual nitrate) or diffusion (ammonium) methods using the following equation: δ^15^N_DON_ = (δ^15^N_TDN_ × [TDN] - (δ^15^N_ammonium_ × [ammonium] + δ^15^N_nitrate_ × [nitrate]))/[DON].

### ^15^N Natural Abundance Method

We used δ^15^N values of soil N (DON, ammonium, and nitrate) in control pots and δ^15^N values of the leaves in a Bayesian isotope-mixing model [Stable Isotope Analysis in R (SIAR)] to estimate the proportional contributions (*f*, %) of the three N forms (DON, ammonium, and nitrate) to total N of each species. The SIAR model uses a Bayesian framework to establish a logical prior distribution (60) for estimating *f* values, and then determines the probability distribution for the *f* values of each soil N source (*f*_DON_, *f_ammonium_* and *f_nitrate_*, presented here as the average probability) to the mixture (δ^15^N_leaves_, in this study). The mixing model considers isotope effects during plant N uptake (^15^𝜀 values hereafter). We used the normalized ^15^𝜀 values for worldwide plants associated with ectomycorrhizae (ECM) and arbuscular mycorrhizae (AM) (-3.2 and -2.0‰, respectively; Craine et al., 2009) to correct the δ^15^N_leaves_ value:

1 = *f*_DON_ + *f*_ammonium_+ *f*_nitrate_;
^∗^δ^15^N_leaves_ = δ^15^N_leaves_ + 3.2‰ (for ECM species *Pinus, Larix*, and *Quercus*);
^∗^δ^15^N_leaves_ = δ^15^N_leaves_ + 2‰ (for AM species *Juglans*);
^∗^δ^15^N_leaves_ = δ^15^N_DON_ × *f*_DON_ + δ^15^N_ammonium_ × *f*_ammonium_ + δ^15^N_nitrate_ ×*f*_nitrate._

The differences in ^15^N uptake rates among the three labeled N forms for each species were tested through one-way analysis of variance. *P*-values < 0.05 were considered to indicate a significant difference among means. Analyses were followed by Tukey’s b *post hoc* test. All statistical analyses were conducted using SPSS 19.0 (SPSS, Inc., Chicago, IL, United States).

## Results

### ^15^N Natural Abundance and N Source Partitioning

**FIGURE 1:**
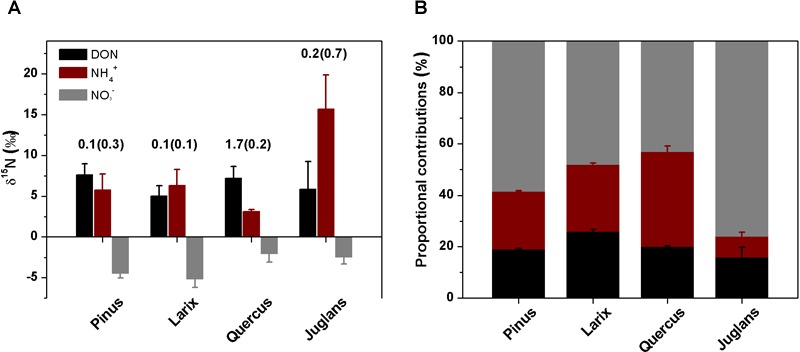
δ^15^N values (**A**, ‰) of soil DON, ammonium, nitrate (bars, means ± SE) and of leaves after corrected for the ^15^𝜀 (numbers above the bars) and the estimated proportional contributions (**B**, %, means ± SD) of the three N forms to plant N nutrition of the four taxa *Pinus, Larix, Quercus*, and *Juglans* using an isotope mixing model (see section “Materials and Methods”).

Before ^15^N tracer addition, plant organs of the four species had δ^15^N values ranging from -3.1 to -0.5‰, with N concentrations ranging from 0.4 to 3.6% (Table 1). The calculated whole plant δ^15^N values ranged from -3.0‰ for *Larix* to -1.1‰ for *Quercus* (Table 1), with *Quercus* having the highest δ^15^N values of leaf, stem, coarse, and fine roots (Table 1). δ^15^N of soil N pools, on the other hand, ranged from 5.0 to 7.2‰ for DON, 3.1 to 15.7‰ for ammonium, and -5.2 to -2.1‰ for nitrate (Figure 1A). DON was the dominant N form within these soils (Table 2), with concentration ranging from 4.4 ± 1.4 to 7.0 ± 0.7 μg g^-1^, followed by nitrate (1.5 ± 0.2 to 3.5 ± 1.3 μg g^-1^) and ammonium (1.2 ± 0.1 to 2.5 ± 0.4 μg g^-1^). The nitrate : ammonium ratios varied between 1.3 ± 0.2 and 2.1 ± 0.4 (Table 2). Soils planted with *Quercus* had the lowest N availability (DON + ammonium + nitrate) after 1 year growth (Table 2), but had highest δ^15^N values of leaf, stem, coarse roots, and fine roots (Table 1).

**Table 1:** N concentration and ^15^N natural abundance within plant organs.

Species	Leaf		Stem		Coarse roots		Fine roots		Whole plant
	[N]%	δ^15^N (‰)	[N]%	δ^15^N (‰)	[N]%	δ^15^N (‰)	[N]%	δ^15^N (‰)	δ^15^N (‰)
*Pinus koraiensis*	2.05 (0.11)	-3.1 (0.5)	0.63 (0.13)	-3.1 (0.3)	1.11 (0.12)	-3.6 (0.1)	1.42 (0.21)	-0.9 (0.3)	-2.8 (0.3)
*Larix keampferi*	2.44 (0.15)	-3.1 (0.2)	0.62 (0.16)	-3.2 (0.1)	1.06 (0.13)	-3.3 (0.2)	1.32 (0.34)	-0.9 (0.4)	-3.0 (0.1)
*Quercus mongolica*	1.75 (0.32)	-1.5 (0.1)	0.35 (0.13)	-1.3 (0.1)	1.07 (0.32)	-0.7 (0.1)	1.32 (0.21)	-0.5 (0.1)	-1.1 (0.2)
*Juglans mandshurica*	3.62 (0.71)	-1.8 (0.8)	0.56 (0.17)	-1.5 (0.5)	1.64 (0.73)	-1.6 (0.6)	1.01 (0.24)	-1.0 (0.8)	-1.6 (0.7)

**Table 2:** Soil pH, dissolved organic nitrogen (DON), ammonium, nitrate, the nitrate to ammonium ratio, plant dry weights and root : shoot ratios of seedlings of the four common tree species in northeast China after 1 year of pot growth.

Species	pH	DON (μg g^-1^)	Ammonium (μg g^-1^)	Nitrate (μg g^-1^)	Nitrate : ammonium ratio	Dry weight (g)	Root : shoot ratio
*Pinus koraiensis*	6.2 (0.2)	6.9 (0.5)	1.6 (0.1)	3.4 (0.9)	2.1 (0.4)	42 (4)	1.1 (0.1)
*Larix keampferi*	6.1 (0.2)	7.0 (0.7)	1.3 (0.1)	2.0 (0.3)	1.5 (0.1)	233 (24)	0.3 (0.1)
*Quercus mongolica*	5.7 (0.1)	4.7 (0.5)	1.2 (0.1)	1.5 (0.2)	1.3 (0.2)	344 (34)	0.8 (0.1)
*Juglans mandshurica*	5.8 (0.1)	4.4 (1.4)	2.5 (0.4)	3.5 (1.3)	1.4 (0.4)	449 (62)	1.1 (0.2)

*Values are presented as means ± SE (*n* = 4).*

Bayesian isotope-mixing model estimates using natural δ^15^N values of soil N (DON, ammonium, and nitrate) in control soils and δ^15^N values of the leaves after correction of the fractionation factor (^15^𝜀) showed that soil nitrate was the major N source for all species, with *f_nitrate_* values ranging from 43 to 76% (Figure 1B), followed by DON (*f*_DON_, 16% ∼26%) and ammonium (*f_ammonium_*, 7 ∼ 37%, Figure 1B).

### ^15^N, ^13^C Uptake and Assimilation

**FIGURE 2:**
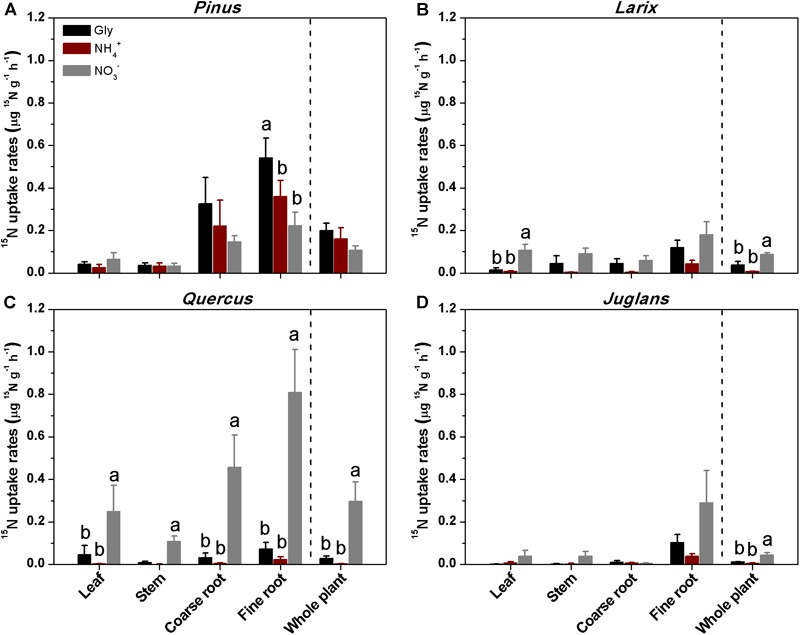
Mean (± 1 SE, *n* = 4) uptake rates of ^15^N by different plant organs and whole plants of the four taxa **(A–D)** at 48 h after soil injection of ^15^N as glycine, ammonium, or nitrate. Different letters above the bars indicate significant differences in uptake rates between N forms for each species (one-way ANOVA, *P* < 0.05).

All four species could access N from the three chemical forms supplied. At 24 h after ^15^N addition, *Pinus* and *Larix* varied widely in ^15^N uptake by coarse and fine roots, and only assimilated small amounts of ^15^N in leaves, which limited our ability to detect N uptake preference in these two species (Supplementary Figure S1). After 48 h of ^15^N tracer addition, plant organs of *Larix, Quercus*, and *Juglans* receiving ^15^${\mathrm{NO}}_{3}^{–}$ treatment had the highest ^15^N uptake rates, followed by those receiving U-^13^C_2_, ^15^N-glycine. Across all plant species and plant organs, ^15^N uptake rates for all three N forms ranged from 0.001∼0.8 μg ^15^N g^-1^h^-1^, with the highest being the uptake of nitrate by *Quercus* fine roots (0.8 ± 0.2 μg ^15^N g^-1^h^-1^), and the lowest being the assimilation of ammonium to *Quercus* stems (0.001 ± 0.001 μg ^15^N g^-1^h^-1^; Figure 2C). Nitrate uptake rates, at 0.006 ∼ 0.8 μg ^15^N g^-1^h^-1^, were 2 ∼ 12 times that of glycine (0.002 ∼ 0.12 μg ^15^N g^-1^h^-1^) and 8 ∼ 85 times that of ammonium (0.001∼0.04 μg ^15^N g^-1^h^-1^). *Quercus* had significantly higher uptake and assimilation rates for nitrate than for glycine or ammonium (*P* < 0.001 for all plant organs and whole plants; Figure 2C).

**FIGURE 3:**
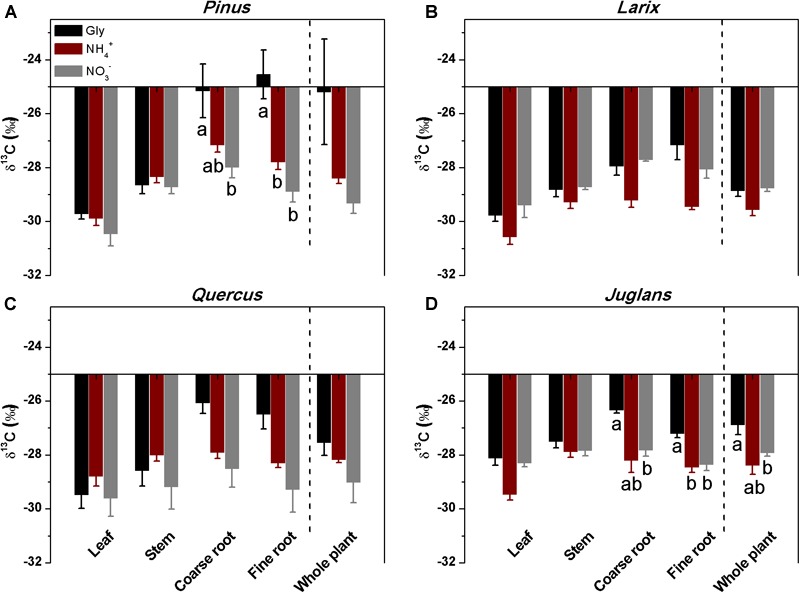
Stable carbon isotope composition (δ^13^C) of different plant organs of the four studied species **(A–D)** that had received mixtures of glycine, ammonium and nitrate, with a single form isotopically labeled. The δ^13^C of the whole plant was calculated from mass-weighted δ^13^C of plant organs (Means ± SE, *n* = 4). Different letters below the bars indicate significant differences in δ^13^C between N forms for each species (one-way ANOVA, *P* < 0.05).

**FIGURE 4:**
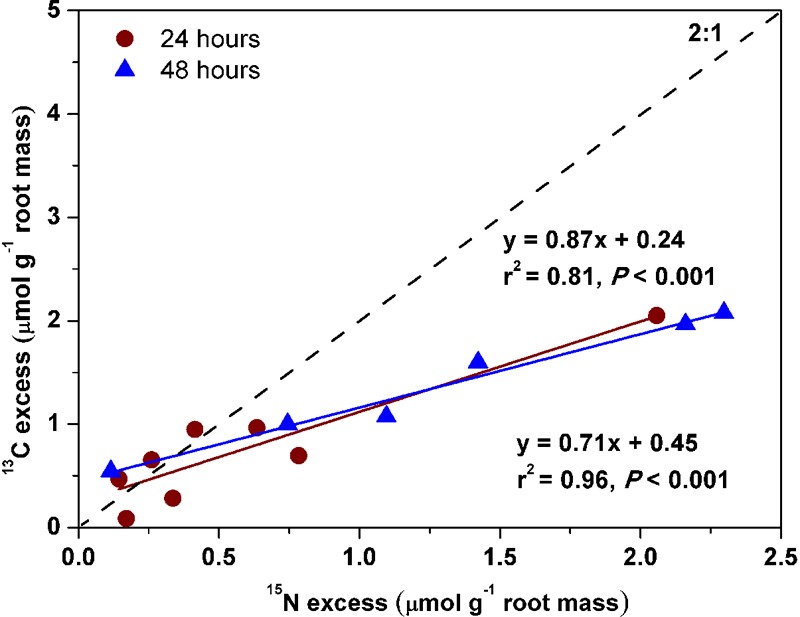
Relationships of ^15^N excess with ^13^C excess in coarse and fine roots of *Pinus koraiensis* that had received U-^13^C_2_, ^15^N-glycine treatment after 24 and 48 h, respectively. The relationships fitted were: ^13^C excess = 0.87 × ^15^N excess + 0.24, *r*^2^ = 0.81, *P* < 0.001 at 24 h and ^13^C excess = 0.71 ×^15^N excess + 0.45, *r*^2^ = 0.96, *P* < 0.001 at 48 h. The expected 2:1 relationship was shown.

In contrast to the other three species, *Pinus* fine roots incorporated most ^15^N from glycine (0.54 ± 0.1 μg ^15^N g^-1^h^-1^), followed by ammonium and nitrate (0.36 ± 0.07 and 0.23 ± 0.06 μg ^15^N g^-1^h^-1^, respectively*; P* < 0.01; Figure 2A). Corresponding to its higher uptake of dual-labeled glycine (U-^13^C_2_, ^15^N-glycine), the coarse and fine roots of *Pinus* were associated with significantly higher δ^13^C values (*P* < 0.05 for both; Figure 3A). The correlation between the ^15^N excess (y) and the ^13^C excess (x) under U-^13^C_2_, ^15^N-glycine treatment had a slope of 0.71 (y = 0.71x + 0.45, *r*^2^ = 0.96, *P* < 0.001) at 48 h after ^15^N tracer addition, indicating that 36% of glycine was derived from intact uptake by *Pinus* roots (Figure 4). In addition, the coarse roots, fine roots, and whole plant of *Juglans* were significantly enriched in ^13^C following U-^13^C_2_, ^15^N-glycine treatment (Figure 3B–D), but the ^15^N excess (y) and the^13^C excess of coarse and fine roots of this species were not significantly correlated (data not shown).

**FIGURE 5:**
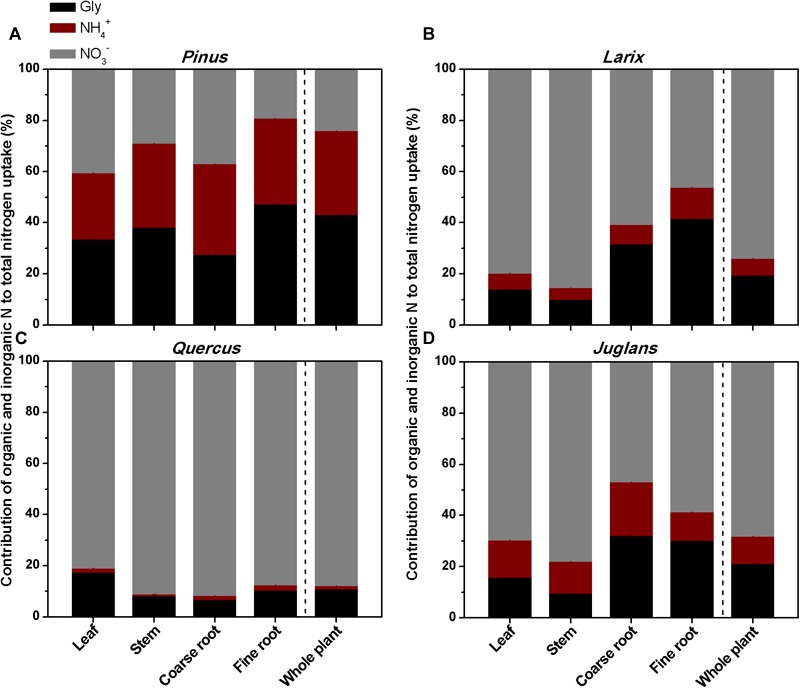
The contribution of glycine, ammonium, and nitrate to total N uptake by different plant organs (leaf, stem, coarse root, fine roots) and whole plant of the four tree species **(A–D)** estimated from the ^15^N labeling experiment.

Overall, for the whole plant, the relative uptake proportion for the three N forms was glycine > ammonium > nitrate (*P* < 0.05) for *Pinus*, and nitrate > glycine > ammonium (*P* < 0.001) for *Larix, Quercus*, and *Juglans* (Figure 5). For *Pinus*, uptake of glycine, ammonium, and nitrate accounted for 43, 33, and 24% of total N uptake, respectively (sum of uptake of the three N forms, calculated with whole plant value) (Figure 5A). In contrast, nitrate uptake contributed 74, 88, and 68% and glycine contributed 20, 11, and 21% to total N uptake by *Larix, Quercus*, and *Juglans*, respectively (whole plant values, Figure 5B–D).

## Discussion

Nutrient acquisition strategies incorporate nutrient uptake rates, mycorrhizal associations, nutrient requirements, and preferences for chemical forms of N, which are analogous to above-ground economic strategies (Wright et al., 2004). In this study, we tracked plant N uptake rates for three N forms from the short-term incorporation of ^15^N-labeled glycine, ammonium, or nitrate by four tree species common in secondary forests and larch plantations in northeast China. These species exhibited different preferences for different N forms, with *Pinus* predominantly accumulating glycine into fine roots and coarse roots, followed by ammonium, while the other three species primarily used nitrate, followed by glycine. These results partly supported our hypothesis, suggesting that tree species within these secondary forests and larch plantations partition N through preferential uptake of different N forms. Nitrate was the most available N form in these sites and was preferred by all species except *Pinus*.

Nevertheless, given that this experiment was done in pots and trees were kept in a greenhouse for a year prior to the study, these results may not reflect what would occur in the field. Being a pot study in greenhouses, the soil within pots might be warmer than field soils, which may enhance both N mineralization and nitrification processes, with the net effects on soil ammonium and nitrate availability not straightforward. Despite this, all species were exposed to the same conditions, so the differences among species are noteworthy.

Moreover, across all four species and all plant organs, the ^15^N uptake rates ranged from 0.001∼0.8 μg ^15^N g^-1^h^-1^. These uptake rates, however, were low comparing with those by plant species from temperate forests in China (root uptake rates, 0.8∼12.8 μg ^15^N g^-1^h^-1^ in Liu et al., 2017), in a wet temperate forest in Tasmania (root uptake rates, 13.5∼173 μg g^-1^h^-1^; Warren and Adams, 2007), and from temperate deciduous forest in eastern North America (22.5 ∼ 255 μg ^15^N g^-1^ fine root h^-1^; Scott and Rothstein, 2011), all of which used hydroponic techniques. Thus these differences might be related to the different labeling methods used by this and other studies. In this study soils within the pots growing tree seedlings were labeled and the whole plants were harvested. Under such conditions, fine roots get access to the N after interceptions by soil microbes and soil abiotic reactions. In contrast, in the hydroponic experiments, where excised or intact roots were subjected to ^15^N labeling solutions to study ^15^N uptake, adsorption by soil particles or soil microbes was minimized.

### Pinus Roots Uptake of Glycine

All four species studied could take up organic N, in the form of glycine, as has been shown for other tree species (Schmidt and Stewart, 1999; Öhlund and Näsholm, 2001; Finzi and Berthrong, 2005). *Pinus koraiensis seedlings* assimilated more glycine than the other species, with glycine contributing 43% to total plant uptake by this species (whole plant value). The overall uptake pattern in *Pinus* was glycine > ammonium > nitrate. Our results agree with studies on *Pinus sylvestris* L. and *Picea abies* L. seedlings growing in pots (peat as medium) showing similar uptake rates of glycine, arginine and ammonium, being 7 to 8 times higher than uptake rates for nitrate (Öhlund and Näsholm, 2001). The relatively high *Pinus koraiensis* fine roots and coarse roots uptake of glycine and ammonium reflected the possibly higher numbers of the transporter proteins for ammonium than for nitrate in the plasma membrane of conifer roots (Kronzucker et al., 1997).

The linear relationship of ^13^C excess with ^15^N excess in *Pinus* (Figure 4; Näsholm and Persson, 2001; Warren and Adams, 2007) indicated that roughly 36% of glycine-N was taken up intact. In this study, the slope of the relationship was 0.71 after 48 h of ^15^N tracer addition, which is less than the expected slope of 2.0, indicating that intact uptake could have been underestimated because post-uptake metabolism of glycine can lead to loss of ^13^C (Warren and Adams, 2007). For example, Hobbie and Hobbie (2012) reported that 25∼38% of added glycine was respired in soils and planktonic systems. Thus, we conclude that the *Pinus koraiensis* seedlings preferentially use glycine and ammonium over nitrate under greenhouse conditions.

We note that we cannot determine the actual chemical form of N that the plant roots took up, nor whether N was directly taken up by plant roots or via mycorrhizal hyphae. Nitrogen transformations can be rapid in temperate forests (Finzi and Berthrong, 2005) and microbially mediated N uptake may have resulted in plants taking up a chemical form of N different from the original ^15^N source. Half-lives of amino acids in temperate soils vary from < 2 to 29 h (Lipson and Näsholm, 2001). Finzi and Berthrong (2005), who examined the cycling of ^15^N-enriched glycine in temperate forest soils in Connecticut, United States, observed rapid immobilization and mineralization of glycine to ammonium by soil microbes, with substantial subsequent nitrification. They considered that sampling at 2 and 5 h following isotope addition might be suitable to assess intact uptake (Finzi and Berthrong, 2005; Gallet-Budynek et al., 2009). Thus, given the rapid turnover time of amino acids in soil (Jones and Hodge, 1999; Finzi and Berthrong, 2005), our sampling time of 48 h may have allowed some glycine to be transformed to ammonium prior to *Pinus* uptake. However, the 36% labeling level of ^13^C derived from glycine indicated considerable intact uptake. The fast turnover of amino acids in soils implies that amino acids may serve as a significant N source even at relatively low concentrations in the soil solution (Kielland et al., 2006).

*Pinus* are obligate symbionts with ectomycorrhizal fungi. At seedling harvest, ectomycorrhizal colonization (fungal sheaths on fine root tips) was observed for each *Pinus* individual (Zhu and Dai, personal observation). Plants with mycorrhizal associations are generally favored over non-mycorrhizal plants in N-limited ecosystems, where most soil N cycling will largely occur in organic forms. Due to the wider enzymatic capabilities of mycorrhizal fungi compared to plant roots (Chalot and Brun, 1998) and because of the small size of hyphae, fungi are better able to penetrate to the sites of organic matter decomposition and compete directly with other microorganisms for the decomposition products (Kong et al., 2017). Thus, it was possible that the detected intact uptake of amino acids may be largely contributed by the mycorrhizal symbiont (Jones, 1999; Näsholm and Persson, 2001).

### Preference of Nitrate by the Other Three Species

In contrast to the *Pinus* species, the other three species, *Larix, Quercus*, and *Juglans* primarily used nitrate, followed by glycine. Such uptake patterns differ from those in temperate forests from China (Donglingshan, Liu et al., 2017), in a wet temperate forest in Tasmania (Warren and Adams, 2007), and two temperate forests in northeastern United States on acidic soils (pH 4; Finzi and Berthrong, 2005), in all of which found ammonium to be preferred by plants over glycine. However, our results resembled that from a sugar maple-white ash site in northeastern United States on dolomitic soil (pH 5.8, Gallet-Budynek et al., 2009), where nitrate and ammonium were preferred over glycine. In addition, previous long-term ^15^N tracer studies on the different fates of deposited ammonium and nitrate in a natural Korean pine and broad-leaved mixed forest at Changbai Mountain, northeastern China, revealed that broad-leaved trees assimilated more nitrate than ammonium over 410 days, with recovery of 16% of added ^15^${\mathrm{NO}}_{3}^{–}$ in aboveground biomass, in contrast to 6% recovery of ^15^${\mathrm{NH}}_{4}^{+}$ (Liu et al., 2016). In the NSF in Qingyuan, plants also retain more nitrate than ammonium after 3 months of tracer addition (Li et al., 2019).

The following potential mechanisms may cause the preferential uptake and assimilation of nitrate by *Larix, Quercus*, and *Juglans* in this study:

The primary reliance on nitrate over glycine and ammonium might be related to the ambient soil availability of these N forms. After 1 year growth of all four species, soils were characterized by higher nitrate than ammonium concentrations, with a nitrate to ammonium ratio of 1.3 ∼ 2.1 (Table 2). Thus, the observed higher uptake of nitrate than glycine or ammonium corresponded well with this predominance of nitrate over ammonium in these soils. In studies of N uptake in tree species along N availability gradients, N uptake rates changed markedly depending on atmospheric N deposition and soil N availability (Högberg, 1997). In this study, we supplied four species of tree seedlings with identical concentrations of ^15^N tracers in the three forms (e.g., glycine: ammonium : nitrate being 1:1:1), and three of the species took up more nitrate than other forms. Considering the dominance of nitrate over ammonium in the ambient soils (nitrate : ammonium greater than 1 : 1; Table 2, Xi et al., 2016), these trees may also take up more nitrate than ammonium in their native environments.

Secondly, the higher uptake capacity for nitrate may reflect the mobility of this ion in the soil. In a temperate grassland soil, nitrate diffused through soil water 36 times faster than glycine and 123 times faster than ammonium (Owen and Jones, 2001), due to the easy sorption of ammonium by negatively charged clay particles and soil organic matter (Barber, 1995). In contrast, glycine is amphoteric and nitrate is negatively charged; they adsorb less readily than ammonium onto exchange sites in soil and both forms are mobile in soil water, often moving to existing root surfaces through transpirational water (Raven et al., 1992; Chapin et al., 2002). Overall, these possible underlying mechanisms to some extent explained the greater reliance of the three species on nitrate over glycine and ammonium, which was also reflected by variations in the pool sizes within the soils.

Thirdly, higher assimilation of ^15^${\mathrm{NO}}_{3}^{–}$ into leaves and stems of the three species *Larix, Quercus*, and *Juglans* (Figure 2) also reflected the characteristic of faster transport of nitrate within plants than amino acids or ammonium (Andrews, 1986). Nitrate can be transported directly from roots to other plant organs before being assimilated into organic compounds via nitrate reductase and the glutamine synthetase/glutamate synthase (GS/GOGAT) system (Andrews, 1986). In contrast, ammonium must be assimilated in roots via the GS/GOGAT system into glutamine before it can be transported throughout a plant (Andrews, 1986). Scott and Rothstein (2011) observed the most ^15^N partition into aboveground organs in the ^15^${\mathrm{NO}}_{3}^{–}$ treatment, the least in the ^15^${\mathrm{NH}}_{4}^{+}$ treatment, and intermediary levels in the ^15^N-amino acid treatments. Greater distribution of nitrate in leaves compared to ammonium and amino acid N cited a faster transport of nitrate out of roots compared to amino acids or ammonium (Persson et al., 2006).

### Implications

Distinguishing the different fates of ammonium and nitrate is a prerequisite to better understand the consequences of increasing N deposition. Deposited ammonium and nitrate are processed differently in a mixed forest in Changbai Moutain (Liu et al., 2016) and in NSF in Qingyuan (Li et al., 2019). In both forests, aboveground plant biomass was a bigger sink for nitrate than for ammonium over 90 days (Li et al., 2019) and 410 days (Liu et al., 2016). Our results from a 48-h labeling experiment provided evidence of plant preferential uptake of nitrate by three species (*Larix, Quercus*, and *Juglans*), corresponding well to the observed different sink strength for ammonium and nitrate by these plants over relative longer terms.

Recently increased proportions of nitrate in N deposition has decreased the mean ammonium : nitrate ratio from 6 in 1980 to 2 in 2010 (Liu et al., 2013). This may increase soil nitrate in these disturbed forest stands, resulting in larger nitrate : ammonium ratios. Our results that *Pinus* assimilated predominantly glycine into fine roots and coarse roots, in contrast to primarily nitrate assimilation by the other three species, *Larix, Quercus*, and *Juglans*, implied that some coniferous trees may compete less well than other species, particularly if they are unable to shift root preference toward increased nitrate availability. Therefore, increasing N deposition may influence the coexistence of plant species and lead to community composition change in these forest stands.

## Data Availability

The raw data supporting the conclusions of this manuscript will be made available by the authors, without undue reservation, to any qualified researcher.

## Author Contributions

YF and FZ conceived and designed the experiments. FZ and LD performed the data acquisition. FZ analyzed the data and wrote the manuscript. YF, EH, KK, XL, GG, SHu, SL, YL, and SHa commented and edited the article.

## Conflict of Interest Statement

The authors declare that the research was conducted in the absence of any commercial or financial relationships that could be construed as a potential conflict of interest.

## Supplementary Material

FIGURE S1 Mean (± 1 SE, *n* = 4) uptake rates of ^15^N by different plant organs and whole plants of the four taxa **(A–D)** at 24 h after soil injection of ^15^N as glycine, ammonium, or nitrate. Different letters above the bars indicate significant differences in uptake rates between N forms for each species (one-way ANOVA, *P* < 0.05).
